# Graduates, 1894

**Published:** 1894-09

**Authors:** 


### Graduates, 1894.

ONTARIO VETERINARY COLLEGE—TORONTO, CANADA.

The annual closing exercises of the Ontario Veterinary College, Temperance Street, were held on Saturday morning, March 24th, 1894, in the Lecture Theatre. Professor Andrew Smith, the worthy Principal, who for the past thirty years and more has so successfully conducted the institution, presided, and was supported on the platform by the following gentlemen:—Mayor Kennedy, Rev. Principal Caven, D.D., J. J. Withrow, President Toronto Industrial Exhibition Association; John Millar, Deputy Minister of Education; J. L. Hughes, Public School Inspector; Dr. Thorburn, Dr. J. T. Duncan, Dr. C. H. Sweetapple, H. J. Hill, Manager Industrial Association; Henry Wade, Secretary Agriculture and Arts Association of Ontario; Rev. C. Campbell, William Cowan, V. S., Galt; Wm. Burns, V. S., President Ontario Veterinary Association; John Akers and others.

The proceedings opened with a few words of welcome from the Chairman, who spoke in most gratifying terms of the continued success of the college, after which a letter of apology for enforced absence was read from the Lieutenant-Governor, who was prevented from being present through domestic bereavement.

The prizes and diplomas were then distributed as follows:

*Degree V.S.* (*Veterinary Surgeon.*)

| | |
|---|---|
| Andrew L. Alton | Oakville. |
| Walter N. Armstrong | London, Ont. |
| Arthur W. Baker | Cortland, N. Y. |
| Henry C. Babcock | College Springs, Iowa. |
| Claude Bailey | Rosemont, Ont. |
| Alfred Barradell | Kettleby, Ont. |
| Levi Porter Beechy | Berlin, Ohio. |
| Christian F. Behner | Grand View, Ill. |
| William R. Bell | St. Louis, Mich. |
| Lincoln L. Bishop | Delavan, N. Y. |
| Judson Black | Yale, Mich. |
| John C. Blackwell | Centralia, Wash. |
| Harry P. Bock | New Ringgold, Pa. |

| | |
|---|---|
| Delbert A. Bonesteel | Frankford, Ont. |
| E. Grant Britton | Guy's Mills, Pa. |
| Thomas Burns | Croghan, N. Y. |
| Joseph Butters | Motherwell, Ont. |
| Andrew J. Battin | Shunk, Pa. |
| Samuel A. Bradley | Louisville, Ky. |
| Archibald B. Campbell | Toronto Ont. |
| Richard Colthurst | Cork, Ireland. |
| Clare V. Connell | Wauseon, Ohio. |
| James Crail | Tipton, Ind. |
| John T. Cunningham | Woonsocket, R. I. |
| Charles F. Curtiss | Parker, S. D. |
| John F. Cline | Glenboro', Man. |
| John S. Dade | Hutchinson, Kansas. |
| Clem. M. Detar | Fryburg, Pa. |
| Richard G. Dingman | Portland, Oregon. |
| Wilber C. Doss | Pittsfield, Ill. |
| James M. Douglas | Dundee, Scotland. |
| Joseph M. Douglas | Hendrum, Minn. |
| Jaroslav J. Drasky | Wilber, Neb. |
| J. W. Dunham | Owatonna, Minn. |
| I. S. Deitrich | Hamburg, Pa. |
| Charles F. Ebner | Ipswich, S. D. |
| Thomas Falconer | Grandin, N. D. |
| Robert O. Fenton | Kingsley, Iowa. |
| B. Fisher | Creston, Iowa. |
| George Fitchett | Pinnebog, Mich. |
| John F. Fitzsimmons | Danbury, Conn. |
| Andrew G. Fortune | Vesta, Ont. |
| Fred L. Foust | Defiance, Ohio. |
| Frank Fowler | Plum Creek, Man. |
| William R. Fullarton | Dubuque, Iowa. |
| George R. Fetherolf | Mahanoy City, Pa. |
| George S. Gates | Oneida, Ill. |
| Otto H. Gebhardt | Alpena, Mich. |
| E. D. Gleason | Carlinville, Ill. |
| Joseph M. Good | Greenwood, Miss. |
| William F. C. Gregg | Barrie, Ont. |
| William J. Glasgow | Springfield, Mass. |
| Francis A. Harsh | Augusta, Ohio. |
| E. W. Heighway | London, Ont. |
| Albert W. Hinman | Braddock, Pa. |

| | |
|---|---|
| Thomas S. Hitch | Griggsville, Ill. |
| John Howie | Barnett, Ont. |
| William F. Heyde | St. Louis, Mo. |
| W. R. Hunter | Lewiston, N. Y. |
| Christian D. Hemmy | Juneau. Wis. |
| Peter I. Johnson | Marion, N. Y. |
| George B. Jones | Nevins, Ill. |
| T. D. Jones | Avoca, Pa. |
| James S. Joyce | Mansewood, Ont. |
| Amandus H. Kistler | Andres, Pa. |
| Albert J. Kline | Ridgeville Corners, Ohio. |
| W. L. P. Knoll | Montrose, Pa. |
| William E. Kreider | Wadsworth, Ohio. |
| John E. Laidlaw | Glenworth, Ont. |
| L. Sherman Lane | Harrisburgh, Ohio. |
| Walter E. Lathrop | Bade Axe. Mich. |
| D. J. McColl | Lucan, Ont. |
| Alexander McConnell | Jarvis, Ont. |
| W. W. McCrae | Steubenville, Ohio. |
| John T. McElroy | Concord, Ont. |
| John McGillicuddy | Watford, Ont. |
| John G. McGuffiin | London, Ont. |
| P. L. McCollum | Lewiston, N. Y. |
| R. J. Marshall | Brandon, Man. |
| Charles H. Eartin | Buffalo. N. D. |
| Harry M. Martin | Townshend, Vt. |
| W. E Martin | Perry, Mo. |
| Robt. E. Monteith | Killarney, Man. |
| Norman Morgan | Strathroy, Ont. |
| Henry H. Muecke | Sioux City, Iowa. |
| Robert Mollance | Kirkcudbright, Scotland. |
| Augustus C. Murphy | Sydney, C. B., Nova Scotia. |
| Sylvester J. Murray | Medo, Minn. |
| S. Forest Musselman | Cynthiana, Ky. |
| Elisha Meyer | Ottawa, Ill. |
| Horace A. Myers | Lodi, Ohio. |
| Sydney D. Myers | Wooster, Ohio. |
| W. M. Molyneaux | Forksville, Penn. |
| John Neville | Decatur, Mich. |
| James Nicholson | Edinburgh, Scotland. |
| Erasmus M. Nighbert | Palmyra. Ill. |
| William C. Orr | Dillon, Montana. |

| | |
|---|---|
| John Henry Penfold | Tweedside, Ont. |
| James W. Price | Reynoldsburg, Ohio. |
| William Pullyblank | Keene, Ont. |
| Sydney J. Rasberry | Hamilton, Ont. |
| William H. Richardson | Watford, Ont. |
| Robert Robb | Newman, Ill. |
| James E. Robertson | West Union, Iowa. |
| Harvey W. Robinson | Guilford, Ind. |
| Z. L. Rogers | East Palestine, Ohio. |
| Richard C. Rolles | Maryborough, Ont. |
| M. B. Rombough | Morden, Man. |
| James D. Ross | Port Dover, Ont. |
| Henry J. Rowe | Sandusky, Ohio. |
| William J. Rooks | Holland, Mich. |
| J. Gill Sallade | New Ringgold, Pa. |
| Charles A. Sankey | Bossevaine, Man. |
| Walter A. Seale | Granby, P. Q. |
| W. J. Shields | Lake Talon, Ont. |
| Ernest H. Shuttleworth | Beaconsfield, Iowa. |
| John A. Smith | Ivan, Ont. |
| Wade H. Smith | Pleasant Corners, Ohio. |
| William H. Smith | Morden, Man. |
| J. Rowe Snively | Lanark, Ill. |
| O. F. Stearns | Windham, Vt. |
| James C. Stevens | De Ruyter, N. Y. |
| E. W. Sunderlin | Auburn, N. Y. |
| Francis Taggart | Wilsonville, Ont. |
| F. Evans Tibbals | Somerset, Ky. |
| Robert Turnbull | Danville, Ill. |
| H. A. Turner | Syracuse, N. Y. |
| Adam R. Van Luven | Cape Vincent, N. Y. |
| George M Walrod | Lake View, Iowa. |
| Frederick A. Walsh | Kingston, Ont. |
| S. Hay Ward | Clandeboye, Man. |
| Le Roy Weber | Rochester, N. Y. |
| H. F. Whaley | San Francisco, Cal. |
| Arthur W. Whitehouse | Laramie, Wyoming. |
| E. J. Whitworth | Lynedoch, Ont. |
| Robert J. Willis | Brampton, Ont. |
| Charles L. Widmeyer | Gretna, Man. |
| C. Otto Wagoner | Akron, Ohio. |
| W. H. Wilkinson | Almont, Mich. |
| John W. Welch | Toronto, Ont. |

PRIZES.

Pathology—A. W. Whitehouse, first prize, silver medal; C. A. Sankey, second prize; W. R. Fullerton and Z. L. Rogers (equal), third prize.

Materia Medica—First prize, A. W. Whitehouse; second prize, C. A. Sankey and G. R. Fetherolf (equal).

Chemistry—N. Morgan, first prize; John F. Fitzsimmons, second prize; T. D. Jones, third prize.

Morbid anatomy—First prize, Arthur W. Whitehouse; second prize, Charles A. Sankey; third prize, Robert Turnbull.

Anatomy—A. W. Whitehouse, silver medal; C. A. Sankey and R. Turnbull (equal), second prize; Judson Black and A. W. Hinman (equal), third prize.

Dissected specimens—A. B. Campbell, gold medal, given by the Toronto Industrial Exhibition Association; A. B. Campbell, second prize, $30; S. A. Bradley, third prize, $20.

Entozoa—Judson Black, S. D. Myers and A. W. Whitehouse (equal), first prize.

Physiology—A. W. Whitehouse, first prize, silver medal; Chas. A. Sankey, second prize; S. H. Ward, third prize.

Best general examination—Chas. A. Sankey, gold medal. Honors—J. Black, G. R. Fetherolf, E. M. Nighbert, Z. L. Rogers, A. W. Whitehouse.

Special prize to the student taking the greatest number of first prizes, A. W. Whitehouse.

THE SPEECHES.

At the conclusion of the prize distribution several of the gentlemen on the platform delivered brief addresses to the students. Mayor Kennedy congratulated the college and the students upon the conclusion of another successful year in the history of the institution. He commented upon the large number of students present from the United States, British Columbia, the Northwest and many other distant points, and declared that this was a strong testimonial to the wide reputation and thorough work of the college.

Rev. Dr. Caven, Principal Knox College, endorsed the Mayor's remarks upon the necessity and value of continuous study. This, he declared, was not only a necessity, but the highest joy. The best moral attainments were nearly always found alongside of the best intellectual attainments; a good student was nearly always a good man. He was satisfied the students before them would do all in their power to elevate and advance their chosen profession.

Public School Inspector J. L. Hughes advised the students to not only keep up with the latest developments in their own profession, but also branch out into other departments. He reiterated and endorsed Henry Irving's advice to the students at Harvard, to nurture and develop their individualism. There was a great personal responsibility attaching to each of them, a responsibility to themselves, their college, their parents, their neighbors, their country and their God.

Mr. J. J. Withrow, President of the Toronto Industrial Exhibition Association, was the next speaker, and was followed by Mr. Millar, Deputy Minister of Education; Dr. Thorburn, Mr. Cowan and Mr. Burns.

---

### THE KANSAS CITY VETERINARY COLLEGE.

The Kansas City Veterinary College held its third annual commencement on March 20th, at the college building, before a large audience. A number of addresses were delivered by members of the faculty and visiting friends.

The following gentlemen graduated:

*Degree D.V.S. (Doctor of Veterinary Science.)*

| | |
|---|---|
| John H. Pitcairn | Minneapolis, Kan. |
| Dillard Ricketts | Moscow, Mo. |
| John Bell | Coalvale, Kan. |
| Emele Pouppirt | Denver, Col. |

The commencement exercises were more elaborate than in the past. The introductory address was delivered by Dr. Sesco Stewart, and the address on the part of the faculty was made by Dr. Robert Sloan. Degrees were conferred by F. L. DeWolf, president of the college. At the conclusion of the commencement programme the faculty, graduates, alumni and their friends joined in a banquet in the lecture room.

Prizes are not given, as we believe that every man will strive to be the best, and that prizes make considerable ill-feeling among students, as some are sure to be disappointed. There is a senior class for next year of fourteen. There is an optional three years' graded course, allowing a student to matriculate for three years, and taking the first year, anatomy, physiology, chemistry, materia medica and dissection, and each year adding the other studies, as may be required.

### OHIO VETERINARY COLLEGE.

The commencement exercises of the Ohio Veterinary College were held April 4th, 1894, at the College Hall, and were well attended by friends of the students and of the institution.

*Degree D.V.S. (Doctor of Veterinary Science)*

was conferred upon the following gentlemen, who had passed the written and oral examinations held by the Faculty:

| | |
|---|---|
| A. H. McGlasson | Constance, Ky. |
| L. H. Crisler | Bullittsville, Ky. |
| Jos. Lake | Portsmouth, Ohio |
| Rudolph Mertz | Reading, Pa. |
| R. H. Hall | Rushville, Ind. |
| Frank Batten | Lexington, Ky. |
| Andrew Niven | Waupaca, Wis. |
| C. N. Sommer | Webster City, Iowa |
| F. D. Rowell | Excelsior Springs, Mo. |
| W. A. Marshall | Harrogate, Tenn. |
| W. T. Campbell | Cincinnati, Ohio |
| H. D. Heywood | Cincinnati, Ohio |
| Harry Wenborne | Buffalo, N. Y. |
| N. A. Kremer | Canaan, Ind. |
| W. H. Lowe | Paterson, N. J. |

### PRIZES.

Prize for best general examination, fine brass stomach and rectal pump, awarded to Andrew Niven, Waupaca, Wis.

Prize in surgery awarded to Frank B. Batten, Lexington, Ky.

Prize for best paper in pathology, W. A. Marshall.

Physiology, first, Andrew Niven.

Chemistry, first, F. D. Rowell, Jos. Lake.

Histology, first, W. A. Marshall.

Theory and practice, first, Andrew Niven.

Hygiene, first, W. A. Marshall.

Ophthalmology, first, Frank B. Batten.

Materia medica, Harry Wenborne.

Honors, R. J. Hall, F. D. Rowell, Jos. Lake, Rudolph Mertz.

### NEW YORK COLLEGE OF VETERINARY SURGEONS.

The thirty-seventh annual commencement of the New York College of Veterinary Surgeons and School of Comparative Medicine was held at Chickering Hall, Wednesday evening, March 21st, 1894.

In the presence of a crowded hall the diplomas were granted to the following:

*Degree V.S.* (*Veterinary Surgeon.*)

| | |
|---|---|
| Sylvester H. Dean | Grand Forks, N. Dak. |
| David B. Dennish | Trenton, N. J. |
| William M. Fleischman | Westchester, N.Y. |
| William Gall | Aberdeen, Scotland |
| Reuben C. Gross | Eastmont, Pa. |
| Elias A. Gruver | Hellertown, Pa. |
| Patrick J. Hogan | Minesville, N.Y. |
| Peter T. Keeley | Waterbury, Conn. |
| John A. Kiernan | Jersey City, N.J. |
| Wesley Massinger | Chalfont, Pa. |
| Elijah Mathews | Jersey City, N. J. |
| Frank A. McCullough | New York City |
| Bruce McKay | New York City |
| William H. Newton | Dunham Basin, N.Y. |
| Russell Osborne | Wilkesbarre, Pa. |
| John C. Petersen | Copenhagen, Denmark |
| Harry C. Shoemaker | Philadelphia, Pa. |
| Alfred J. Tuxill | Auburn, N. Y. |
| Edward E. Vaughn | Browns Mills, N. J. |

### PRIZES.

John A. Kiernan, Jersey City, gold medal, for best general examination.

Silver medal, for best general junior examination, W. E. A. Wiemann, Brooklyn.

*Special mention*, E. A. Manuel.

Comstock prize, pocket case of instruments, for best anatomical specimen, John C. Petersen, Copenhagen, Denmark.

Jenkins prize, Veterinary books, for the best written and defended thesis, Reuben C. Gross, Eastmont, Pa.

The Buntin prize, case of surgical instruments, for best senior examination in materia medica, John A. Kiernan, Jersey City.

The White memorial prize, case of instruments, for the best practical examination, conducted by a committee composed of Dr. Thomas Giffen, chairman, Dr. Rush S. Huidekoper, Dr. Herbert Neher, awarded to Bruce McKay, New York City.

A banquet was held at the Columbia with seventy-five covers.

### CHICAGO VETERINARY COLLEGE.

The eleventh annual commencement exercises of the Chicago Veterinary College were held Thursday, March 22d, at the Grand Opera House. A large audience, friends of the students and faculty, assembled to witness the exercises. Sixty-five young men received their diplomas. The platform was occupied by the faculty of the college, among the members of which were: President R. J. Withers, M. D. V. S.; A. H. Baker, M. D. C.; Joseph Hughes, M.R.C.V.S.; F. Ellingwood, M.D ; A. S. Alexander, F.H.A.S ; C. E. Sayre, M.D.D.V.S.; J. F. Ryan, M.D.C. The proceedings were opened with an address from President R. J. Withers, who outlined the course of study that had been followed by the successful students, and stated that of the number of graduates the large number of thirty-one passed with honors, whose names are as follows:—J. S. Anderson, M. T. Bernard, G. A. Bond, C C. Brown, A. Collasowitz, P. L. Darcey, G. L. Dixon, G. R. Flowers, D. P. Frame, E. S. Fry, J. H. Gain, T. J. Heter, E. N. Hutchinson, D. E. Kinsella, G. W. Loveland, A. J Mauland, J. H. McLain, W. A. Myers, A. S. Nesbitt, W. H. Netherland, C. J. Rhodes, G. L. Simon, J. D. Sprague, C. E. Stewart, H. L. Stewart, Wm. Thompson, R. H. Treacy, C. A. White, J. W. White, P. J. Wilkinson, A. S. Withers. After the address by the president, the class arose from the body of the theatre and took positions on the platform, where the president conferred the degree M.D.C. on its members, and each received his diploma, after which they returned to their seats, and were addressed by the Hon. John G. Shortall, President of the Illinois Humane Society. Mr. Shortall having finished his address the prizes were distributed. Wm. Thompson received the prize in anatomy and also the prize in materia medica, he having the highest standing in both of these subjects. P. J. Wilkinson received the prize for highest standing in theory and practice. After the prizes were distributed, numerous and beautiful floral tokens, sent by friends of the graduating students, were distributed to various members of the class. The valedictorian, G. R. Flowers, M.D.C., read the valedictory address. Dr. Arthur R. Reynolds, City Health Commissioner, then delivered the doctorate address. The proceedings closed with farewell remarks by the president.

The following are the members of the graduating class and their present locations:

*Degree M.D.C.* (*Doctor of Comparative Medicine.*)

| | |
|---|---|
| J. S. Anderson | Seward, Neb. |
| W. F. Andrews | Meadville, Pa. |
| M. T. Bernard | Serena, Ill. |
| J. Biggs | Hubbard, Ia. |
| G. A. Bond | Vicksburg, Mich. |
| C. J. Britton | Crawfordsville, Ind. |
| C. C. Brown | Rockville, Ind. |
| Wm. F. Brownlee | Clinton, Ia. |
| J. J. Bruemleve | St. Louis, Mo. |
| H. J. Campbell | Paxton, Ill. |
| P. A. Carlson | Mason City, Neb. |
| C. A. Clinton | Havelock, Ia. |
| A. Collasowitz | Cincinnati, O. |
| P. L. Darcey | Chicago, Ill. |
| G. L. Dixon | Racine, Wis. |
| S. H. Ellery | Brimfield, Mass. |
| G. R. Flowers | Lyndonville, N. Y. |
| D. P. Frame | Colorado Springs, Colo. |
| E. S. Fry | Naperville, Ill. |
| J. H. Gain | Ada, Minn. |
| F. T. Gaskin | Plainfield, Ill. |
| T. J Heter | Sterling, Kan. |
| T. H. Hicks | Milbank, S. D. |
| J. P. Hornig | Rockfield, Wis. |
| F. Hunt | Paxton, Ill. |
| E. N. Hutchinson | Odell, Ill. |
| H. Jones | Williamsville, Ill. |
| J. F. Jones | Wellington, Kan. |
| H. J. Kannal | Rensselaer, Ind. |
| R. Kerr | Whitewater, Wis. |
| D. E. Kinsella | Wyoming, Ill. |
| R. Kuoni | Sauk City, Wis. |
| W F. Lazear | Derby, Ia. |
| G. W. Loveland | Colebrook, Conn. |
| W. Matz | Bellevue, O. |
| A. J. Mauland | Chicago, Ill. |
| W. E. McBain, Jr. | Toledo, O. |
| B. C. McClintock | Meadville, Pa. |
| E. A. McCullough | Caledonia, Wis. |
| J. H. McLain | Inkster, N. D. |
| D. H. Miller | Norton, Kan. |

| | |
|---|---|
| W. A. Myers | Wenona, Ill. |
| A. S. Nesbitt | Maroa, Ill. |
| W. H Netherland | Louisville, Ky. |
| C. O. Netherton | Gallatin, Mo. |
| J. W. Otto | Magnolia, Ill. |
| J. N. Piatt | Boonville, Ind. |
| C. J. Rhodes | Roscoe, Ill. |
| A. L. Robinson | Winsted, Conn. |
| O. Rydell | Minneapolis, Minn. |
| S. K. Shenck | Salina, Kan. |
| G. L. Simon | Huntertown, Ind. |
| J. D. Sprague | Owensville, Ind. |
| C. E. Stewart | Oakley, Ia. |
| H. J. Stewart | Chicago, Ill. |
| H. L. Stewart | Oakley, Ia. |
| J. W. Styles | Peotone, Ill. |
| Wm. Thompson | Calumet, Ill. |
| F. P. Tobin | Chicago, Ill. |
| R. H. Treacy | Steele, N. D. |
| B. F. Ward | Braidwood, Ill. |
| C. A. White | Chicago, Ill. |
| J. W. White | Roberts, Ill. |
| P. J. Wilkinson | Delafield, Wis. |
| A. S. Withers | Chicago, Ill. |

---

### AMERICAN VETERINARY COLLEGE.

The nineteenth annual commencement of the American Veterinary College was held at Chickering Hall, March 20th, 1894. The hall was crowded as usual with friends of the college and members of the Alumni. The programme was enlivened by the 7th regiment band. The following gentlemen received diplomas:

*Degree D.V.S.* (*Doctor of Veterinary Surgery.*)

| | |
|---|---|
| Albert Francis Abbott | Dover, N. H. |
| George Cornelius Bretherton | New York |
| John Russell Bacon | Danbury, Ct. |

| | |
|---|---|
| Hugh Edward Clark | Cornwall-on-Hudson, N. Y. |
| Chauncey Leonard Chase | Berlin, Ohio |
| James Shaw Cattanach, Jr. | New York |
| Augustus Henry Drucker | New York |
| Michael Joseph Doyle | New York |
| Harry Stillwell Field | Hempstead, N. Y. |
| Oscar Fausner | New York |
| William Rowan Fleming | Brooklyn, N. Y. |
| John Oliver George, A. B., A. M., | Weaversville, Pa. |
| Hamilton Gutmann | Easton, Pa. |
| John Joseph Hayes | New York |
| Jacob P. Helmer | Scranton, Pa. |
| Richard Wilkins Hewitt | Bridgeton, N. J. |
| Harry G. Hoover | Sterling, Ill. |
| John Kent | New York |
| Newton C. Lazarus | Allentown, Pa. |
| George Leich | Brooklyn, N. Y. |
| James Lorimer Lindsay | New York |
| Patrick John McGuiness | Newark, N. J. |
| Francis Joseph McCaffrey | Brooklyn, N. Y. |
| Charles McCulloch, Jr. | Howardsville, Va. |
| James Masterton | Biggar, Lanarkshire, Scotland |
| Charles Philip Martin | New York |
| William Brundage Moorhouse | Tarrytown, N. Y. |
| Robert Flanders Moore | Pocatello, Idaho |
| Ira Chasey Mattatall | West Tatamagouche, N. S. |
| Bryce Mars | New York |
| Harry Stanislaus O'Neill | Jamaica, N. Y. |
| Oscar T. Porzer | Brooklyn, N. Y. |
| Joseph Forman Roser | Maysville, Ky. |
| John Joseph Riordan | Beverly Farms, Mass. |
| Frederick Luman Ray | Leonia, N. J. |
| Albert E Schnable | Indianapolis, Ind. |
| Wm. Christian Siegmund | Baltimore, Md. |
| Harry Clifton Terry | Torresdale, Pa. |
| Henry Otto Wolters | New York |
| Andrew Ward | Peacedale, R. I. |
| Christian Henry Westphal | San Francisco, Cal. |
| William Watkins Yard, Jr. | New York |

PRIZES.

Trustees Prize.—A gold medal for the best general examination, to W. Christian Siegmund, D.V.S.

Faculty Prize.—A gold medal for the best practical examination before a committee of practitioners, appointed from N. Y. City and Brooklyn, to Richard Wilkins Hewitt, D.V.S.

Alumni Prize.—A set of veterinary books for the second best general examination, to William Rowan Fleming, D.V S.

Senior Anatomy Prize.—A case of Instruments for the best dried and mounted preparation, illustrating Descriptive or Surgical Anatomy, to Robert Flanders Moore, D.V.S.

College Class Prize. —A case of instruments for the best paper read and discussed before the Class Association, to John Oliver George, A.B., A.M., D V S.

Junior Anatomy Prize.—For the best examination in Junior Anatomy, to Mr. W. Grüzman.

Class Officers.—President, Harry S. Field ; Vice-President, Michael J. Doyle ; Secretary, George H. Hoey ; Treasurer, J. Harry Hutchinson ; Historian, John Kent.

Executive Committee.—Chairman, John O. George ; Bryce Mars, Oscar Fausner, Henry O. Wolters, William R. Fleming, Albert E. Schnable, Albert F. Abbott, John J. Hayes, William B. Moorhouse, Frederick L. Ray.

Marshall, Harry G. Hoover.

